# Functional divergence and regulatory network of the type VI secretion system in *Vibrio parahaemolyticus*

**DOI:** 10.1128/jb.00378-25

**Published:** 2025-12-17

**Authors:** Xiaowen Wang, Pu Yao, Lei Liu, Yiquan Zhang

**Affiliations:** 1 Department of Pharmacy, Southwest Hospital, Army Medical University (Third Military Medical University) 12525 , Chongqing, China; 2 Department of Transfusion Medicine, General Hospital of Central Theater Command, Wuhan, Hubei, China; 3 Department of Clinical Laboratory, Nantong Third People’s Hospital, Affiliated Nantong Hospital 3 of Nantong University, Nantong, Jiangsu, China; Southern University of Science and Technology, Shenzhen, Guangdong, China

**Keywords:** *V. parahaemolyticus*, T6SS, effector, function, regulation

## Abstract

*Vibrio parahaemolyticus* is a significant marine pathogen causing gastroenteritis and wound infections in humans. Its pathogenicity is mediated by key virulence factors, including the type VI secretion system (T6SS). This review comprehensively synthesizes current knowledge on the functional divergence and regulatory networks of T6SSs in *V. parahaemolyticus*, with emphasis on T6SS1 and T6SS2. T6SS1, enriched in clinical isolates, is activated under high-salt and warm conditions and primarily facilitates antibacterial competition and adhesion to human epithelial cells in strains such as RIMD2210633. T6SS2, nearly ubiquitous across strains, operates optimally under low-salt/low-temperature conditions and regulates adhesion, biofilm formation, motility, macrophage autophagy induction, and virulence as demonstrated in strains including RIMD2210633 and SH112. Both systems deploy diverse effectors (e.g., Tme1, PoNe, and RhsP) targeting membrane integrity, DNA, or peptidoglycan. Their expression is intricately controlled by environmental cues (e.g., salinity, temperature, and metal ions), stress responses (e.g., antibiotics, ethanol, and curcumin), quorum sensing regulators (e.g., AphA and OpaR), and transcriptional factors (e.g., H-NS, TfoY, and CalR). Strain-specific functional variations highlight the complexity of T6SS biology. Understanding these mechanisms offers insights for developing anti-virulence strategies against *V. parahaemolyticus* infections.

## INTRODUCTION

*Vibrio parahaemolyticus* is a significant marine pathogen with a distinct distribution pattern, thriving naturally in marine environments such as seawater, brackish estuaries, and coastal mudflats. It is also prevalent in seafood including shellfish, shrimp, crabs, and fish, which can be contaminated during harvesting, handling, or storage (1). Infections from this bacterium pose a substantial health risk. Consumption of contaminated seafood frequently causes acute gastroenteritis, characterized by a rapid onset of nausea, vomiting, abdominal pain, and watery diarrhea that can result in severe dehydration (2). Additionally, skin infections may occur through wound contact with contaminated water or seafood, presenting with redness, swelling, pain, exudate, and potentially cellulitis (2). In immunocompromised individuals, *V. parahaemolyticus* can invade the bloodstream, leading to life-threatening septicemia marked by high fever, chills, and hypotension, a condition that requires urgent treatment (2).

The pathogenicity of *V. parahaemolyticus* is mediated by an array of virulence factors (3). While hemolysins such as thermostable direct hemolysin (TDH) and TDH-related hemolysins are well-established major contributors to pathogenesis (4), the existence of pathogenic strains lacking genes encoding these hemolysins underscores the critical involvement of additional mechanisms (5). Among these, the type VI secretion system (T6SS) has emerged as another key virulence determinant (6). Pangenomic analysis revealed that the genome of *V. parahaemolyticus* contains four distinct T6SS gene clusters, designated T6SS1, T6SS2, T6SS3, and T6SS4 (7). While T6SS3 and T6SS4 are rare and their functions remain largely unexplored, T6SS1 and T6SS2 are the most widespread and best-characterized (7). Notably, T6SS1 distribution is enriched in clinical isolates, whereas T6SS2 is nearly universal in both clinical and environmental strains (7). Functionally, T6SS1 contributes to both antibacterial activity and general cell adhesion, whereas T6SS2 primarily mediates specific cell adhesion and facilitates biofilm formation (8–10). The secretion activity of both systems is differentially regulated by environmental factors: high-salt/30°C conditions upregulate T6SS1, whereas low-salt/23°C conditions enhance T6SS2 activity (11). The expression of T6SS1 and T6SS2 is also modulated by quorum sensing (QS), specific transcriptional regulators, and metal ions (12–14). Given the complexity and critical roles of T6SS1 and T6SS2 in the pathogenicity and environmental adaptation of *V. parahaemolyticus*, this review systematically summarizes their diverse functions and intricate regulatory network, explores their role in adaptive evolution, and outlines future research directions and potential applications.

## TYPICAL T6SS IN BACTERIA

**Fig 1:**
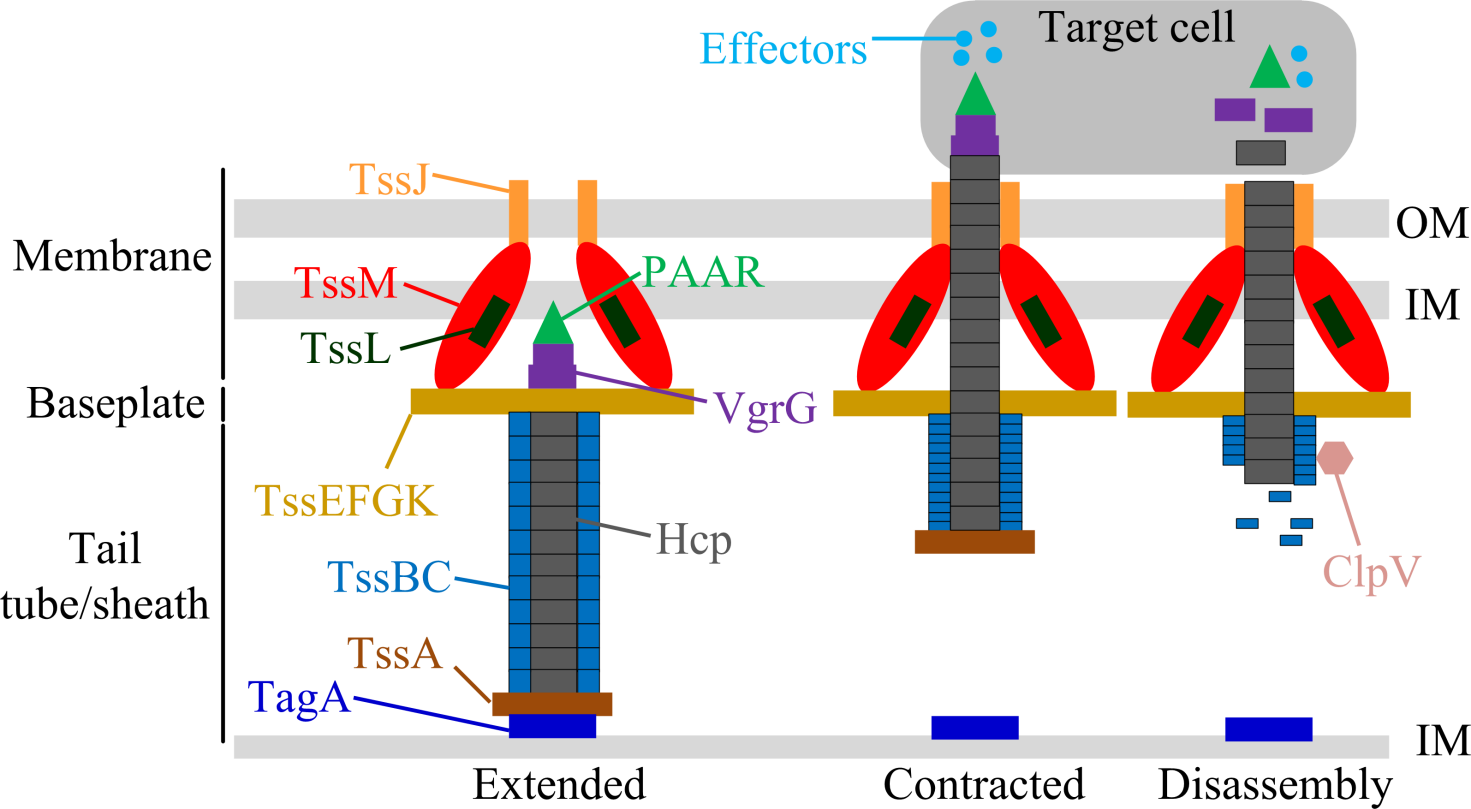
Schematic diagram of the T6SS structure. The T6SS is a contractile nanomachine composed of three core complexes. The membrane complex (TssJ, TssL, and TssM), embedded in the inner (IM) and outer (OM) membranes, provides a structural foundation. The baseplate complex (including TssE, TssF, TssG, TssK, and the trimeric VgrG-PAAR spike) serves as an assembly platform. The contractile tail tube/sheath complex consists of an inner Hcp tube surrounded by a TssB-TssC sheath. During firing, sheath contraction propels the VgrG-PAAR spike and Hcp tube, loaded with effectors, into a target cell. Following contraction, the ClpV ATPase disassembles the sheath for recycling.

T6SS is a nanoscale, multi-protein injection apparatus with three core components: the membrane complex, baseplate complex, and tail tube/sheath complex (Fig. 1) (15). Anchored in the inner membrane and extending to the outer membrane, the membrane complex forms a ~ 30 nm × 20 nm pentameric “rocket” structure from TssJ (outer membrane lipoprotein), TssL, and TssM (inner membrane protein) (16). Their cytoplasmic domains link to the baseplate, stabilizing the T6SS. The baseplate, comprising over 60 peptides (including TssE, TssF, TssG, TssK, and VgrG), acts as a structural platform and loading dock (17). VgrG, a key component of the baseplate, forms trimeric spikes often tipped with PAAR proteins to pierce target cells (17, 18). It also anchors the tail tube/sheath complex to it and serves as a platform for loading effectors onto the apparatus. The tail tube/sheath complex features an inner Hcp hexameric tube that serves as a potential conduit for some effectors and as a structural component, surrounded by a contractile sheath formed from TssB-TssC dimers (19). Assembly of the tail is facilitated by TssA. Distal end stabilization is mediated by TagA specifically in long-term residency systems (15, 17). Contraction of the sheath drives the VgrG-PAAR tip into target cells and injects the effectors. Following contraction, the ClpV ATPase recognizes exposed TssC N-termini to disassemble and recycle the sheath for reassembly (19).

It is important to note that the above description represents the canonical T6SS model, primarily based on studies in bacteria such as *V. cholerae* (20). However, *V. parahaemolyticus* T6SS1 and T6SS2 exhibit notable deviations from this general model. For instance, T6SS2 contains two TagF homologs—an uncommon feature—while lacking the canonical PpkA/PppA pair and TagA anchoring protein depicted in the schematic (7). These differences imply distinct architectures and regulation mechanisms that are discussed in subsequent sections.

## GENETIC STRUCTURE AND EXPRESSION PATTERNS OF T6SS1 AND T6SS2

**Fig 2:**
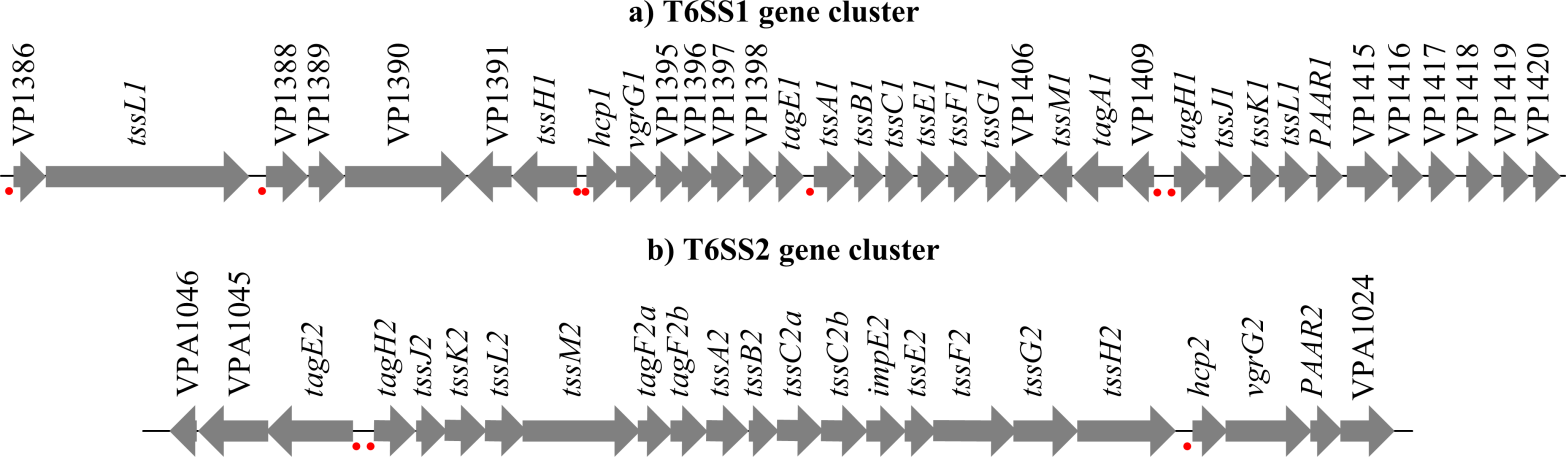
The T6SS genetic clusters in *V. parahaemolyticus* RIMD2210633. Gene clusters for (**a**) T6SS1 (VP1386-1420) on the large chromosome and (**b**) T6SS2 (VPA1025-1046) on the small chromosome are shown. Thick arrow lines denote genes, with the arrow direction indicating transcription direction. Red dots mark the first gene in an operon. The genetic organization highlights the multi-operonic structure of these systems, with T6SS1 being notably larger and more complex than T6SS2.

Pangenome analysis reveals that *V. parahaemolyticus* carries four distinct T6SS gene clusters, among which T6SS1 and T6SS2 are the most widely distributed (7). The genome of the reference strain RIMD2210633 consists of two chromosomes—a large chromosome (~3.29 Mb) and a small one (~1.88 Mb)—together encoding more than 4,800 genes (21). Each chromosome harbors one T6SS cluster: T6SS1, located on the large chromosome, comprises 35 genes organized into at least seven operons (VP1386-1420); T6SS2, on the small chromosome, consists of 22 genes grouped into three operons (VPA1025-1046) (Fig. 2) (21). Notably, in strain RIMD2210633, T6SS1 secretion and expression are induced under high-salt and warm (30°C) conditions, whereas T6SS2 is activated under low-salt and low-temperature (23°C) conditions (11). Importantly, neither system secretes detectable effectors at 37°C in this strain (11). This suggests that T6SS action on host cells during infection may involve mechanisms other than effector protein secretion, potentially relying on other virulence factors (11). The capacity of T6SS1 and T6SS2 to secrete effector proteins—and the identity of such effectors—at the human body temperature of 37°C remains important topics for future investigation.

## BIOLOGICAL FUNCTION OF T6SS1 AND T6SS2

The T6SS exhibits significant functional diversity and contributes directly to various biological processes, including bacterial pathogenicity, metal ion transport, biofilm formation, and bacterial interactions (9, 11, 22). In *V. parahaemolyticus*, T6SS1 and T6SS2 perform overlapping yet distinct roles (Table 1). A comparative analysis of these two systems in *V. parahaemolyticus* RIMD2210633 reveals their differential involvement in host cell adhesion (8). Specifically, deletion of *icmF1* and *hcp1*—key components of T6SS1—impairs adhesion to both HeLa and Caco-2 cells. In contrast, deletion of *icmF2* and *hcp2*, which are central to T6SS2, affects adhesion only to HeLa cells. This functional specialization may stem from differences in the cell surface receptors recognized by each system (8).

**TABLE 1:** Comparison of the functions of T6SS1 and T6SS2

Functional categories	T6SS1 function	T6SS2 function
Adhesion	Promotes adhesion to HeLa and Caco-2 cells (8).	No role in Caco-2 adhesion (8).
Antibacterial activity	Selectively inhibits competitors lacking the system, enhancing environmental adaptability (11).	Deletion of *tssL2* in strain SH112 reduces this activity against *E. coli* DH5α (9).
Effect on macrophages	Direct evidence for this function is lacking.	Deletion of *vgrG2* (a structural gene) reduces cAMP levels in RAW264.7, triggering non-cytotoxic autophagy (23).
Biofilm formation	No relevant research has been found.	Inhibits biofilm formation in strain SH112, while promotes low-salt biofilm formation in strain RIMD2210633 (9, 10).
Motility	No relevant research has been found.	Promotes swimming and swarming motility in strain SH112 (9).
Virulence regulation	May contribute indirectly via antibacterial activity; direct evidence is lacking.	Deletion of *tssL2* in strain SH112 reduces cytotoxicity toward Caco-2 cells and suppresses key virulence factors such as T3SS1 (9).

However, all these findings were derived from *in vitro* experiments. Since T6SS1 and T6SS2 in *V. parahaemolyticus* RIMD2210633 do not secrete effectors at 37°C (11), it remains unclear whether they mediate bacterial adhesion to host cells during actual infection. Interestingly, the T6SS structure itself can facilitate transient, high-affinity contact with target cells. Through sheath contraction, the needle tip (VgrG–PAAR) establishes direct physical contact with the target cell membrane, forming a transient intercellular connection. This contact may enhance bacterial persistence or effector delivery even in the absence of canonical secreted effectors, though direct experimental evidence for a dedicated adhesion role is limited (11, 24).

Moreover, in *V. parahaemolyticus* strain HZ, deletion of *vgrG2*—a key structural component of T6SS2—lowers cAMP levels in RAW264.7 macrophages and triggers autophagy without causing cytotoxicity (23). T6SS1, on the other hand, selectively inhibits the growth of competing bacteria that lack this system, thereby improving environmental fitness (11, 25).

Notably, functional variation exists among different strains. In strain SH112, TssL2 is essential for T6SS2 structural integrity, Hcp2 secretion, and antibacterial activity (9). It also plays important roles in biofilm formation, motility, virulence, and host immune responses (9). Deletion of *tssL2* in SH112 impaired antibacterial activity against *Escherichia coli* DH5α, reduced adhesion and cytotoxicity toward Caco-2 cells, diminished swimming and swarming motility, and downregulated key virulence factors such as T3SS1, while enhancing biofilm formation (9). Conversely, in strain RIMD2210633, IcmF2 promotes low-salt biofilm formation by time-dependently regulating extracellular DNA, protein synthesis, and c-di-GMP levels, involving *cpsA*, *mfpA*, and c-di-GMP metabolism-related genes (10). These differences highlight that T6SS function varies considerably even within the same species, likely due to variations in gene composition and regulatory networks, though the precise mechanisms require further investigation.

In summary, current evidence suggests that T6SS1 primarily mediates antibacterial activity and adhesion to HeLa and Caco-2 cells, whereas T6SS2 mainly facilitates adhesion to HeLa cells, induces RAW264.7 macrophage apoptosis, exhibits anti-*E*. *coli* activity, and regulates biofilm formation, motility, and virulence. These diverse functions are executed by a repertoire of system-specific effectors, which are delivered into target cells to disrupt critical cellular processes. However, these functions are not universally conserved across all strains, as illustrated by the differences between SH112 vs RIMD2210633.

The functional diversity of T6SS1 and T6SS2 is further illustrated by the strain-specific variations summarized in Table 2.

**TABLE 2:** Characteristics of key *V. parahaemolyticus* strains mentioned in this review^*a*^

Strain	Source	T6SS1	T6SS2	T6SS3	T6SS4	Key findings related to T6SS	References
RIMD2210633	Clinical	+	+	-	-	Reference strain; T6SS1 activated at high-salt/30°C (antibacterial activity, adhesion); T6SS2 activated at low-salt/23°C (adhesion, biofilm, virulence); no effector secretion at 37°C.	(7, 10, 11, 21, 26)
SH112	Clinical	+	+	-	-	TssL2 essential for T6SS2 integrity, antibacterial activity, biofilm formation, motility, virulence; downregulates T3SS1.	(7, 9)
HZ	Clinical	+	+	-	-	VgrG2 of T6SS2 reduces cAMP and induces non-cytotoxic autophagy in RAW264.7 macrophages.	(7, 8, 23)
12-297/B	Shrimp pathogen	+	+		-	Encodes T6SS1 effector V12_14465 (PoNe DNase) with immunity protein V12_14460 (PoNi).	(7, 27)
BB22OP	Clinical	+	+	-	-	T6SS1 effector Tme1 (membrane disruption) with immunity Tmi1; T6SS2 effector RhsP (nuclease); T2LipB effector identified.	(7, 28, 29)
A3	Environmental	+	+	-	-	*hcp1* downregulated at 3% NaCl; *hcp2* downregulated at 1.5% NaCl.	(7, 30)
D4	Environmental	+	+	-	-	Both *hcp1* and *hcp2* downregulated at tested salinities.	(7, 30)
Vp_AHPND_ 123	Shrimp pathogen	?	?	?	?	QseC negatively regulates T6SS; deletion upregulates 22 T6SS genes.	(31)

^*a*^ A plus sign indicates the presence of the gene locus, a minus sign indicates the absence, and a question mark indicates that it is unknown.

## T6SS EFFECTORS

The biological functions of T6SS1 and T6SS2 described above are mediated by a diverse array of secreted effector proteins. Effectors are highly diverse and can be classified based on their structural features or biological functions/targets. Structurally, they often contain characteristic domains such as the Marker for type sIX effectors (MIX) domain, which is a key hallmark of many effectors with polymorphic C-terminal toxin domains, or belong to specialized families like Rearrangement hotspot (Rhs) proteins (32). Functionally, effectors are classified as antibacterial or anti-eukaryotic based on their target and the presence of cognate immunity genes. Their C-terminal toxin domains confer activities such as pore-forming, phospholipase, nuclease, peptidoglycan hydrolase, or protease functions (32). In most *Vibrionaceae* genomes (approximately 36.8%), one to two MIX effectors are encoded, while only a small fraction (around 3.5%) contains more than four (32). In *V. parahaemolyticus*, the repertoire of effectors exhibits considerable strain-specific variation. The following sections summarize the characterized effectors based on their primary functional classes.

### Nuclease effectors

Nuclease effectors target DNA or RNA of recipient cells, leading to cell death. In *V. parahaemolyticus* strain 12-297/B, a novel accessory T6SS module includes *vgrG1b* and its downstream effector/immunity (E/I) pair, V12_14465 (effector)/V12_14460 (immunity protein) (Table 3) (27). The effector V12_14465 contains an N-terminal FIX domain and a C-terminal PoNe DNase toxin, which belongs to the PD-(D/E)xK superfamily. Its DNase activity depends on conserved aspartate residues (e.g., D335) and is neutralized by the DUF1911-domain immunity protein V12_14460 (PoNi) through direct interaction (27). Genes encoding PoNe toxins are typically found adjacent to their corresponding *poNi* genes (27).

**TABLE 3:** Characterized T6SS E/I pairs in *V. parahaemolyticus* strains

Effector	Immunity	Belonging	Strain	Reference
Nuclease effectors				
V12_14465 (PoNe)	V12_14460 (PoNi)	T6SS1	12-297/B	(27)
VPA1263	Vti2	T6SS1	RIMD2210633	(33, 34)
VP1415	VP1416	T6SS1	RIMD2210633	(33)
RhsP (VP1517/T2Rhs-Nuc)	RhsPI (VP1518)	T6SS2	RIMD2210633, BB22OP	(28, 35)
Membrane-disrupting effectors				
Tme1 (VPBB_RS15030)	Tmi1 (VPBB_RS15035)	T6SS1	BB22OP	(29)
Peptidoglycan-targeting effectors				
VP1390	VP1389	T6SS1	RIMD2210633	(24)
VP1388	VP1389	T6SS1	RIMD2210633	(24, 33)
Other/uncharacterized effectors				
T2LipB (VP0626)	Non-detailed	T6SS2	BB22OP	(28)

In the reference strain RIMD2210633, secretome analysis identified VPA1263 as a T6SS1-secreted effector (33). VPA1263 possesses an N-terminal MIX domain, a LysM domain, a PyocinS domain, and an HNH nuclease toxin domain (Table 3) (34). Its MIX domain is essential for T6SS secretion and toxicity, and the HNH domain is responsible for degrading DNA both *in vitro* and *in vivo* to induce prey lysis (34). Another T6SS1 effector in RIMD2210633, VP1415, contains a PAAR-repeat protein, a predicted AHH nuclease effector domain, and a MIX domain and is essential for T6SS1-dependent killing (33).

For T6SS2, the strain RIMD2210633 secretes RhsP (VP1517), a large, folded effector that undergoes autoproteolytic cleavage (35). The C-terminal fragment (RhsPC) contains a WHH nuclease domain (35). The nuclease activity of RhsPC is neutralized by its cognate immunity protein, RhsPI (VP1518), which binds to RhsPC and masks its DNA-binding site (35). In strain BB22OP, the RhsP homology (annotated as T2Rhs-Nuc) is also conserved T6SS2 effector and is essential for T6SS2 activity, suggesting a potential role in the quality control of T6SS2 assembly or effector loading (Table 3) (28).

These nuclease effectors constitute a key antibacterial armament of T6SSs, directly contributing to the bacterial competition activity described in Section 4, particularly for T6SS1.

### Membrane-disrupting effectors

This class of effectors compromises the membrane integrity of target cells. In *V. parahaemolyticus* BB22OP, VPBB_RS15030 (Tme1) and VPBB_RS15035 (Tmi1) form a confirmed T6SS1-dependent antibacterial E/I pair (Table 3) (29). Tme1 requires a functional T6SS1 for secretion and belongs to the widespread Tme family in Proteobacteria (29). Tme effectors localize to the bacterial periplasm and kill target cells by disrupting membrane integrity, as evidenced by loss of membrane potential and increased permeability. Their cognate Tmi immunity proteins, which contain transmembrane domains, specifically counteract this toxicity (29).

### Peptidoglycan-targeting and other effectors

Some effectors target bacterial cell wall components. In strain RIMD2210633, the VP1388-VP1390 operon encodes a unique binary effector module for T6SS1 (24, 33). In this module, VP1388 acts as a co-effector and VP1390 as the toxin, while VP1389 provides immunity (24). VP1388 contains an OmpA_C peptidoglycan binding domain and a MIX domain (24, 33). Functionally, VP1390 causes periplasmic toxicity leading to cell swelling and lysis in *E. coli* (24). VP1388 alone is nontoxic but is essential for the secretion of VP1390 (24). The two proteins interact directly and exhibit interdependent secretion, as deletion of either gene blocks secretion of the other despite normal expression (24). Both proteins coprecipitate with VgrG1, indicating that they are loaded as a complex onto the T6SS apparatus (24).

Other conserved effectors have been identified whose precise mechanisms remain less clear. For instance, T2LipB (VP0626) is a conserved T6SS2-secreted effector in strain BB22OP, though its detailed function is not yet characterized (Table 3) (28).

### Chaperones and effector loading

The proper assembly and secretion of T6SS effectors often require specific chaperones. A key example is the DUF2169 protein, which is crucial for assembling a non-canonical T6SS spike complex. This complex involves VgrG and PIPY-domain-containing proteins, which serve as an alternative version of the typical PAAR-domain spike tip protein (36). DUF2169 stabilizes the VgrG-PIPY complex by binding to the PIPY domain, preventing exposure of its hydrophobic surface and subsequent aggregation (36). In *V. parahaemolyticus*, the absence of DUF2169 causes defects in VgrG1 secretion and T6SS1 assembly, while complementation restores function (36). Biochemical analyses confirm a direct DUF2169-PIPY interaction, and mutations in critical DUF2169 residues disrupt this binding, impair bactericidal activity.

## REGULATION OF T6SS IN *V. PARAHAEMOLYTICUS*

The expression and activity of T6SS in *V. parahaemolyticus* are governed by a complex, multi-layered regulatory network. This network integrates diverse factors, which can be broadly categorized into: (i) abiotic environmental cues (e.g., temperature, salinity, metal ions), (ii) biotic and chemical stimuli (e.g., antibiotics, host-derived molecules, competitor presence), and (iii) intracellular regulatory circuits (e.g., QS, transcriptional and post-transcriptional regulators). These inputs converge to fine-tune T6SS deployment in a system-specific and often strain-dependent manner, optimizing fitness across vastly different environmental and host niches. Rather than a simple list of regulators, this section is organized to first describe key environmental inputs, then dissect the core intracellular circuitry, and finally integrate how these layers interact to coordinate T6SS expression in response to complex stimuli.

**Fig 3:**
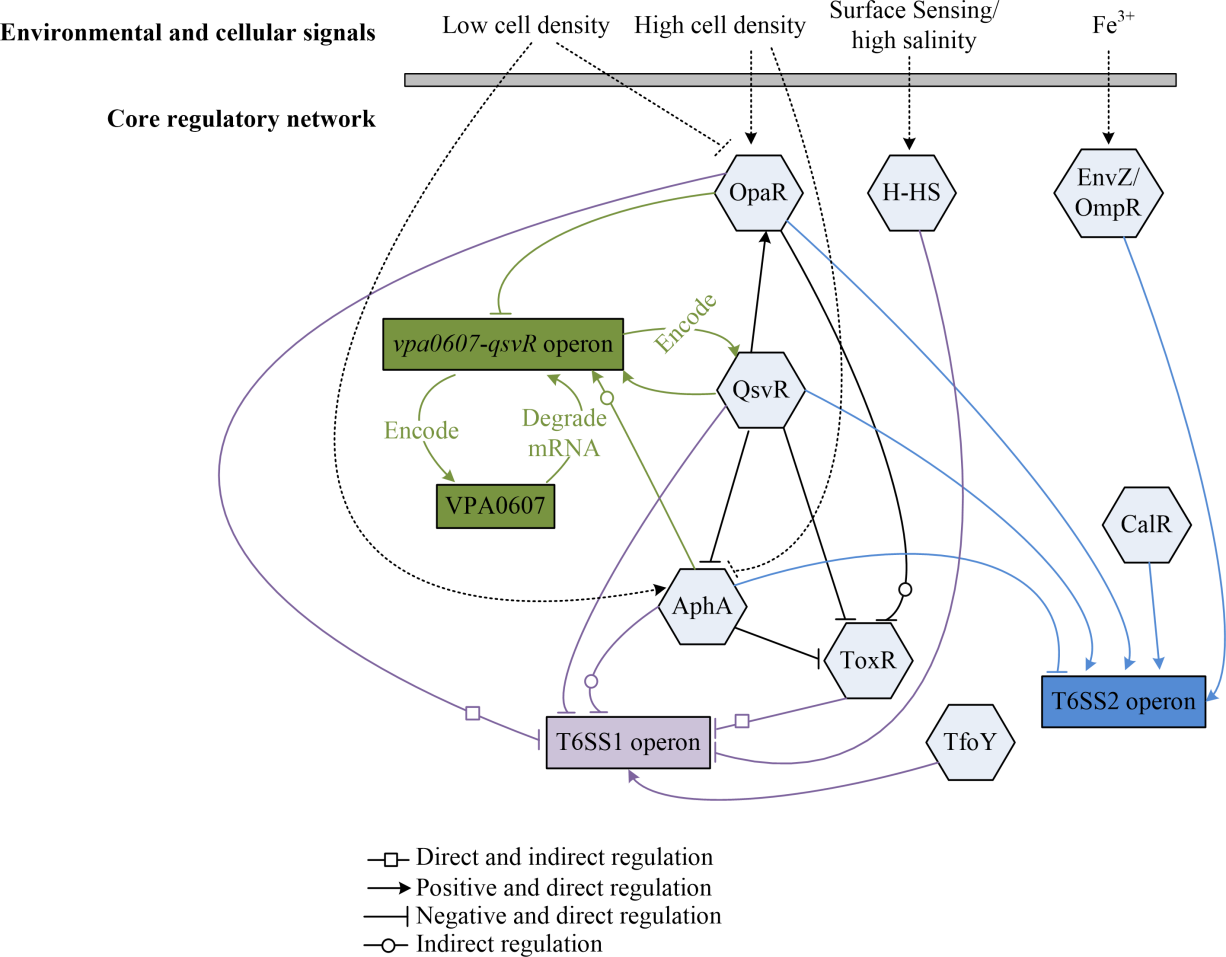
Integrated regulatory network of T6SS1 and T6SS2 in *V. parahaemolyticus*. This schematic summarizes the multi-layered regulation, incorporating environmental cues (salinity and metal ions), cellular signals, and key regulators (QS-related and others). Environmental signals are sensed and transduced through specific regulators (e.g., H-NS for salinity and EnvZ/OmpR for Fe^3+^) and other transcriptional factors (e.g., CalR) to differentially modulate T6SS1 and T6SS2 expression. Dashed lines indicate indirect and complex effects.

To provide a clearer overview of the integrated regulatory framework, we have summarized the major environmental cues and their effects on T6SS1 and T6SS2 in Table 4 and depicted the core regulatory network in Fig. 3. These additions aim to synthesize the multi-layered complexity and facilitate a high-level understanding of how multiple signals converge to modulate T6SS activity.

**TABLE 4:** Summary of major environmental cues regulating T6SS1 and T6SS2 in *V. parahaemolyticus*

Environmental cue	Effect on T6SS1	Effect on T6SS2	Strain(s)	Key regulators/mechanisms
Salinity	Activated under high salt (e.g., 3% NaCl) (11)	Activated under low salt (e.g., 1.5% NaCl) (11)	RIMD2210633	H-NS (represses T6SS1 under low salt) (26)
Temperature	Activated at 30°C (11)	Activated at 23°C (11)	RIMD2210633	Unknown thermosensor; H-NS independent (26)
Metal ions				
Mg^2+^	Suppressed (13)	Suppressed (13)	RIMD2210633	Unknown
Ca^2+^	Suppressed (e.g., VP1409) (37)	No direct effect reported	RIMD2210633	Unknown
Fe^3+^	No direct effect reported	Activated via EnvZ/OmpR (38)	RIMD2210633	EnvZ senses Fe^3+^, phosphorylates OmpR to bind T6SS2 promoters (38)
Chemical stressors				
Ampicillin	Downregulated (39)	Upregulated (39)	RIMD2210633	Unknown
Chloramphenicol	Marginally suppressed (e.g., *hcp1*) (40)	Preferentially inhibited (40)	RIMD2210633	Unknown
L-arabinose	Upregulated (e.g., VP1420) (41)	Downregulated (41)	RIMD2210633	Unknown
Ethanol	Upregulated (42)	Downregulated (42)	RIMD2210633	Unknown
Curcumin	Mixed regulation (downregulates VP1387, upregulates others) (43)	Upregulated (e.g., *hcp2*) (43)	RIMD2210633	Unknown
Host-derived molecules				
DOC	Activated (44)	No direct effect reported	RIMD2210633	Direct activation of T6SS1 transcription and apparatus assembly (44)
TDC	Weakly activated (44)	No direct effect reported	RIMD2210633	Less effective than DOC (44)
Physiological state				
Biofilm formation	Downregulated in early and mature biofilms (45)	Downregulated, especially in mature biofilms (45)	RIMD2210633	Unknown
Wrinkly colonies	Downregulated (46)	Downregulated (46)	RIMD2210633	Unknown
Swarm colonies	Upregulated in released cells (47)	No direct effect reported	RIMD2210633	Unknown
Host infection	Upregulated from 36 h post-infection in *C. elegans* (48)	Upregulated from 36 h post-infection in *C. elegans* (48)	RIMD2210633	Unknown
QS	Peaks at OD_600_ ~ 0.6-0.8 (49)	Increases with cell density (50)	RIMD2210633	Complex QS circuit integrating AphA, OpaR, QsvR, and ToxR (49–52)

### Salinity and temperature

The activity of T6SS in *V. parahaemolyticus* is highly dependent on specific environmental conditions, with distinct regulatory patterns observed between systems and strains. Under steady-state conditions, T6SS1 is activated at a higher temperature (30°C) and high salinity, as evidenced by the expression and secretion of Hcp1 (11). In contrast, T6SS2 is activated at a lower temperature (23°C) and low salinity, correlating with Hcp2 expression and secretion (11). Importantly, this conditional activation requires sustained steady-state conditions rather than abrupt shifts. While abrupt thermal shocks (e.g., to 4°C, 15°C, or 42°C) induce widespread differential gene expression, virulence-associated genes, including those encoding T6SS components, remain largely unaffected (53). Beyond temperature, salinity also serves as a key regulatory signal, though its effects are strain-dependent. For instance, in strain A3, *hcp1* is downregulated at 3% NaCl, while *hcp2* is downregulated at 1.5% NaCl (30). This system-specific response to salinity contrasts with strain D4, in which both *hcp1* and *hcp2* are significant downregulated at both tested salinity levels (30). Although the precise thermosensing and osmosensing mechanisms remain incompletely elucidated, these environmental cues are likely integrated through specific transcriptional regulators. A key example is the nucleoid-associated protein H-NS, which acts as a key repressor of T6SS1 under low-salt conditions; this repression is relieved under high salt, suggesting that salinity modulates H-NS DNA-binding activity (26). Furthermore, the persistence of T6SS1 inhibition at 37°C in an *hns* deletion mutant strongly implies the existence of other, unidentified temperature-dependent regulators (26).

In summary, T6SS regulation is characterized by its responsiveness to specific steady-state environmental cues, coupled with a remarkable degree of system-specific and strain-dependent heterogeneity. This complex conditional regulation reflects an ecological adaptation. As a bacterium inhabiting dynamic aquatic and host environments, fine-tuning T6SS activity according to prevailing temperature and salinity may represent an optimal strategy for energy conservation and precise deployment of virulence factors in appropriate niches, although the exact sensory and signaling mechanisms remain to be fully elucidated.

### Metal ions

Metal ions serve as critical signals for T6SS regulation, often with opposing effects. High Mg^2+^ concentration (55 mM) significantly inhibits the expression of most T6SS1-related genes (31.4%) and T6SS2-related genes (87.0%) in *V. parahaemolyticus* RIMD2210633 (13). This inhibition is associated with the downregulation of key T6SS structural and effector genes—including *hcp1*, *tssA1*, and *tssB1* (T6SS1), as well as *hcp2*, *tssM2*, and *tagH2* (T6SS2)—through Mg^2+^-mediated modulation of global gene expression networks (13). Similarly, 4 mM Ca^2+^ inhibits expression of the T6SS1 gene *vp1409* (37), indicating that both divalent cations broadly suppress T6SS genes, albeit with distinct target specificities.

Notably, the regulatory influence of metal ions on T6SS in *V. parahaemolyticus* RIMD2210633 extends beyond Mg^2+^ and Ca^2+^. For instance, Fe^3+^ activates T6SS2 expression via the EnvZ/OmpR system (38). In this pathway, Fe^3+^ directly binds the periplasmic domain of the histidine kinase EnvZ, triggering autophosphorylation at His232 and activating EnvZ (38). Activated EnvZ then phosphorylates the response regulator OmpR, which subsequently binds to the promoters of T6SS2 genes (e.g., *hcp2* and *tssH2*) to enhance their transcription (38). This Fe^3+^-dependent activation contrasts with the suppressive effects of Mg^2+^ and Ca^2+^, underscoring the presence of metal ion-specific regulatory circuits for T6SS in *V. parahaemolyticus*. Additionally, T6SS regulation in this pathogen involves other systems, such as BarA/UvrY (discussed in below), which may interact with metal ion signaling to fine-tune T6SS function in response to environmental cues (48).

Mechanistically, Mg^2+^ and Ca^2+^ suppress T6SS gene expression through multiple pathways. Transcriptomic data reveal that Mg^2+^ downregulates genes encoding T6SS structural components while altering the expression of 18 c-di-GMP metabolism genes (13). The consequent reduction in intracellular c-di-GMP levels—known to inhibit biofilm formation and virulence gene expression—may indirectly suppress T6SS transcription (13, 54). Mg^2+^ also modulates the expression of over 80 putative regulators (e.g., OpaR), some of which likely target T6SS promoters (13, 49, 50). In the case of Ca^2+^, suppression of *vp1409* is accompanied by downregulation of five c-di-GMP metabolism genes and 24 regulatory genes (e.g., CalR, VtrA), suggesting pathways that partially overlap with yet are distinct from those of Mg^2+^ (37).

The physiological relevance of Mg^2+^-mediated T6SS suppression is evident in both environmental and host contexts. Mg^2+^ is the second most abundant cation in seawater, the natural habitat of *V. parahaemolyticus*, and its concentration fluctuates with factors such as coastal runoff, salinity changes, and algal blooms (13, 55). In host environments, Mg^2+^ levels vary: within the intestinal lumen—where *V. parahaemolyticus* colonizes—Mg^2+^ concentrations are influenced by diet (e.g., high-Mg^2+^ foods like leafy greens or seafood) and host renal/intestinal Mg^2+^ homeostasis (56). Excess Mg^2+^ levels (e.g., Mg^2+^-supplemented diets) may serve as a “habitat signal” prompting *V. parahaemolyticus* to downregulate T6SS—an energy-intensive virulence system—when active host infection is unnecessary. Beyond its role in membrane integrity (Mg^2+^ helps maintain ribosomal stability and supports membrane repair in *V. parahaemolyticus* [57]), this ion acts as a key environmental sensor that helps balance metabolic cost and virulence potential. However, enhanced bacterial adhesion to HeLa cells under high Mg^2+^ conditions—despite strong suppression of T6SS2 gene expression—suggests the existence of T6SS2-independent or compensatory adhesion mechanisms (13). This observation reveals complex regulatory dynamics in which divalent cations decouple virulence gene expression from functional phenotypes, likely as an adaptation to optimize bacterial fitness across diverse environmental and host niches.

### Chemical stressors and host-derived molecules

*V. parahaemolyticus* employs its two T6SS systems for distinct ecological and pathogenic functions: T6SS1 primarily mediates interbacterial competition to enhance environmental fitness and host intestinal colonization, whereas T6SS2 is associated with host cell adhesion, biofilm formation, and virulence (8, 10, 11). Throughout its life cycle, this bacterium encounters diverse stressors, including antibiotics, disinfectants (or food additives), and host-derived molecules (e.g., bile acids). The differential regulation of T6SS1 and T6SS2 under these stress conditions represents a key adaptive strategy for maintaining survival, colonization, and pathogenicity.

This regulatory divergence is particularly evident during antibiotic exposure. In strain RIMD2210633, ampicillin (400 µg/mL), a β-lactam antibiotic, downregulates T6SS1 core structural genes—*vp1413* (encoding the baseplate subunit TssK) and *vp1414* (encoding the secretion system component IcmH)—while upregulating five key T6SS2 genes, including *hcp2*, *tssF2*, and *tssB2* (involved in toxin translocation, baseplate construction, and contractile sheath assembly, respectively), (39). This pattern suggests an “energy trade-off” mechanism under antibiotic stress: by repressing T6SS1-mediated interbacterial competition, the bacterium may reallocate resources to maintain T6SS2-dependent host adhesion, thus preserving colonization potential. In contrast, sublethal chloramphenicol exhibits an opposing preference, predominantly inhibiting most T6SS2 genes (e.g., *tssM* and *tssL*) while only marginally suppressing a few T6SS1 genes like *hcp1* in strain RIMD2210633 (40). As chloramphenicol inhibits protein synthesis, its preferential suppression of T6SS2 may reduce the production of non-essential virulence components, thereby conserving energy for resistance adaptation.

In response to disinfectants and food-related additives, T6SS regulation generally enhances bacterial competitiveness. As demonstrated in strain RIMD2210633, L-arabinose (1%) upregulates the key T6SS1 gene *vp1420* while downregulating most T6SS2 genes (41). Similarly, ethanol (1.5%), a common disinfectant, significantly upregulates 17 genes within the T6SS1 gene clusters and downregulates five T6SS2 genes (42). This consistent trend—activating T6SS1 to eliminate competing microbes while inhibiting T6SS2 to reduce adhesion-related energy costs—likely facilitates bacterial survival and dispersal under disinfectant stress. Curcumin (25 µg/mL), however, exhibits a mixed regulatory pattern: it downregulates only *vp1387* of T6SS1 but upregulates three other T6SS1 structural genes, while simultaneously upregulating eight T6SS2 genes, including *hcp2* (43). This dual regulation may allow the bacterium to balance competitive prowess and adhesive capacity when confronting natural antimicrobials.

Notably, host gut-derived molecules demonstrate clear functional specificity in T6SS regulation. Deoxycholate (DOC), a secondary bile acid modified by host intestinal commensal bacteria, significantly activates T6SS1 transcription and promotes the expression, secretion, and apparatus assembly of VgrG1 in strain RIMD2210633 (44). Strains pre-incubated with DOC exhibit a 10-fold increase in T6SS1-dependent killing of prey bacteria such as *V. natriegens*, whereas taurodeoxycholate (TDC), another bile acid, shows a weaker stimulatory effect (44). This response is directly relevant to intestinal colonization: as a “signal molecule” in the gut, DOC triggers T6SS1 activation, enabling *V. parahaemolyticus* to outcompete resident commensals and secure a niche—reflecting the bacterium’s sophisticated adaptation to the host microenvironment.

In summary, the distinct regulatory responses of T6SS1 and T6SS2 to antimicrobial agents versus host gut signals reveal a refined adaptive paradigm. Antibiotics and disinfectants primarily induce a functional “rebalancing,” where the bacterium modulates both systems to conserve energy and mitigate toxicity, often sacrificing interbacterial competition or adhesion. In stark contrast, host gut molecules like DOC act as precise “activation signals” for T6SS1, strategically deploying this system to eliminate competitors and establish a colonization niche. This clear functional specialization underscores that T6SS regulation is not a generic stress response but a tailored strategy, finely calibrated to the nature of the specific challenge—whether a chemical threat or a biological opportunity within the host.

### Physiological state and functional trade-offs

The expression of T6SS is highly dependent on the physiological state of *V. parahaemolyticus* (45–47). In strain RIMD2210633, both T6SS1 and T6SS2 genes are significantly downregulated during the early stages of biofilm formation (45). This downregulation can be logically explained by the need for energy conservation and the promotion of biofilm stability: in early biofilm development, *V. parahaemolyticus* prioritizes energy allocation toward synthesizing structures essential for initial attachment and matrix formation, such as the polar flagellum, pili, and exopolysaccharides (45). Since the T6SS machinery—specialized for interbacterial competition (T6SS1) or host cell and surface adhesion (T6SS2)—is not essential during early biofilm establishment, its downregulation avoids unnecessary energy expenditure. Furthermore, reduced T6SS activity helps prevent premature interbacterial conflicts that could disrupt the nascent biofilm, thereby supporting community stability (45).

Notably, T6SS2 downregulation becomes more pronounced in mature biofilms (45), which likely reflects its functional association with adhesion: T6SS2 is involved in *V. parahaemolyticus* adhesion to host cells or surfaces (8, 10). In mature biofilms, where cells are embedded within the extracellular matrix and active adhesion is no longer a limiting factor, decreased T6SS2 expression corresponds to a reduced need for adhesion mechanisms (45). Similarly, most T6SS1 and T6SS2 genes are significantly downregulated in wrinkled colonies compared to smooth ones (46). This coincides with reduced cell adhesion (associated with T6SS2) and antimicrobial activity (a core function of T6SS1) (46, 58), further supporting the notion that T6SS activity is reduced in the wrinkled phenotype—characterized by enhanced biofilm stability and decreased reliance on active competition or surface adhesion (46).

In contrast, swarm colonies of *V. parahaemolyticus* RIMD2210633 continuously release a distinct cell type in which T6SS1 protein expression is significantly higher than in planktonic cells (47). As swarm colonies mature, T6SS1 expression in these released cells increases progressively (47). This expression pattern highlights a specialized role of T6SS1 in interbacterial interactions: given well-documented antibacterial activity of T6SS1, its elevated expression equips released cells to outcompete other bacteria more effectively for limited resources and space when colonizing new environments, such as chitin surfaces or host-associated niches. This competitive advantage directly facilitates the dissemination and successful establishment of *V. parahaemolyticus* across diverse ecological niches.

### Host infection

T6SS1 and T6SS2 are crucial for *V. parahaemolyticus* RIMD2210633 infection of *Caenorhabditis elegans*. In the early infection stage, gene expression of T6SS1 and T6SS2 is repressed, but it is significantly upregulated from 36 h post-infection, implying a role for T6SS in the infection process (48). Both Δ*vipA1* (T6SS1 mutant) and Δ*clpV2* (T6SS2 mutant) exhibit significantly reduced pathogenicity compared to wild type, indicating both systems are required for full virulence (48). *C. elegans* has been widely used as a model host to study bacterial pathogenesis, including infections caused by *Vibrio* species such as *V. cholerae* and *V. vulnificus* (59, 60). Importantly, key virulence factors identified in *C. elegans*, including type III secretion system one and T6SS2, have also been shown to contribute to virulence in mammalian hosts (23, 61), supporting the relevance of the *C. elegans* model for understanding *V. parahaemolyticus* pathogenesis across different hosts, including fish, shrimp, and humans.

### QS-related regulators

The QS system, a widespread bacterial communication mechanism, regulates diverse physiological processes including virulence and biofilm formation (62). In *V. parahaemolyticus*, at low cell density (LCD), low autoinducer levels promote LuxO phosphorylation, activating *qrr* sRNA (Qrr1-5) expression, which enhances AphA translation and inhibits OpaR translation (63). AphA regulates downstream pathways, including those involved in virulence and biofilm formation (63). At high cell density (HCD), high autoinducer levels cause LuxO dephosphorylation, halting Qrr sRNA production; as a result, AphA is not produced, and OpaR is translated (63–65). OpaR regulates downstream targets, including its own gene and *aphA* (63). Therefore, AphA and OpaR function as master QS regulators (64, 65).

In strain RIMD2210633, the *vpa0607-qsvR* operon encodes QsvR and VPA0607 (51). VPA0607 degrades its mRNA to inhibit QsvR post-transcriptionally (51). ToxR controls biofilm formation, motility, and virulence (66, 67). Transcription of *vpa0607-qsvR* and *toxR* is cell density-dependent, peaking at LCD and during the LCD-to-HCD transitions, suggesting QS regulation (52, 67). At LCD, AphA indirectly inhibits *toxR* but activates *vpa0607-qsvR* (51, 67). At HCD, OpaR directly inhibits *vpa0607-qsvR* and indirectly inhibits *toxR*, whereas QsvR directly inhibits *vpa0607-qsvR*, *aphA*, and *toxR* but activates *opaR* (51, 52, 67). Hence, QsvR, ToxR, AphA, and OpaR form a complex regulatory network that controls gene expression.

**Fig 4:**
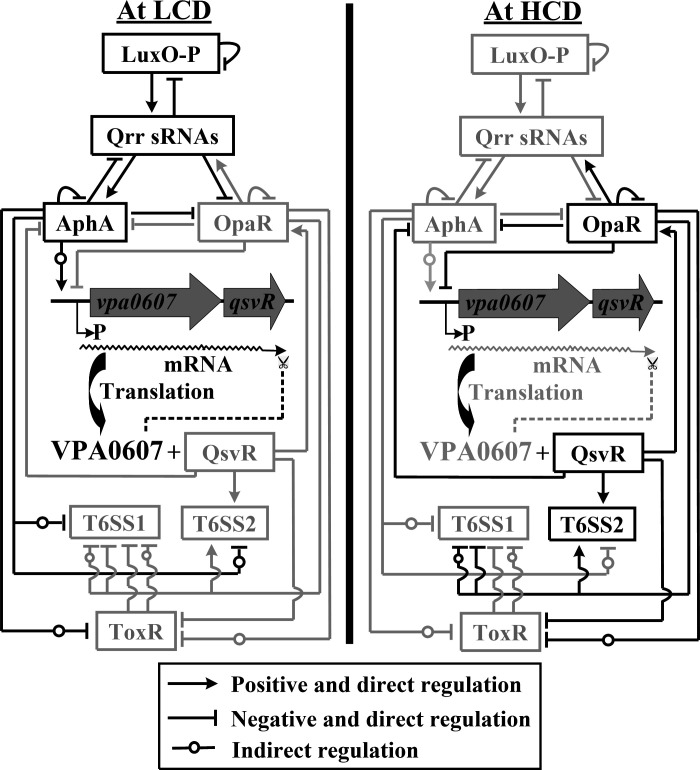
Schematic diagram of QS-dependent T6SS expression in *V. parahaemolyticus* RIMD2210633. The model integrates the master QS regulators AphA (LCD) and OpaR (HCD) with intermediate regulators QsvR and ToxR to differentially control T6SS1 and T6SS2. Wavy lines represent mRNA; dashed lines terminating in scissor symbol indicate post-transcriptional degradation; gray fonts and lines denote the absence or weakening of regulatory relationships; black lines and fonts indicate the presence of regulatory relationships.

For T6SS1 in strain RIMD2210633, at LCD, AphA indirectly inhibits the operons *vp1388-1390*, *vp1393-1406*, *vp1400-1406*, and *vp1409-1407* (49). At HCD, OpaR indirectly inhibits *vp1393-1406* and directly inhibits the other three operons, while QsvR directly inhibits *vp1388-1390* and *vp1393-1406* (49, 68). During the LCD-to-HCD transition, ToxR directly inhibits *vp1400-1406* and *vp1409-1407* and indirectly represses the others (49). Consequently, T6SS1 expression peaks at OD_600_ of 0.6 to 0.8 (67). For T6SS2 in strain RIMD2210633, at LCD, AphA indirectly downregulates the operons *vpa1027-1024*, *vpa1043-1028*, and *vpa1044-1046* (50). At HCD, OpaR and QsvR bind their promoters to activate transcription; thus, T6SS2 expression increases with cell density (50, 69). Additionally, CqsA-mediated QS (CMQS) inhibits T6SS2 by downregulating OpaR, as demonstrated in Δ*cqsA*, where T6SS2 proteins and mRNA increase but decrease in Δ*opaR* and Δ*cqsA*Δ*opaR* (70). CMQS also inhibit T6SS2-mediated adhesion via OpaR (70). In summary, QS-associated regulators coordinate the temporal expression of T6SS1 and T6SS2 (Fig. 4) to enable adaptive regulation.

### Other important regulatory factors

Beyond direct environmental sensing, a core set of conserved transcriptional regulators forms the backbone of T6SS control, often sharing functional themes with other vibrios while exhibiting species-specific peculiarities. As mentioned, H-NS is a key silencer of T6SS1, with its repression being lifted by high salinity and surface sensing (Table 5) (26, 71). The persistent thermosensitivity of T6SS1 even in the absence of H-NS points to a complex, multi-factorial control of temperature response that is a critical area for future research.

**TABLE 5:** Other key regulatory factors of T6SS in *V. parahaemolyticus*

Regulatory factors	Effect on T6SS	Strain	Key observations	References
Positive regulators				
TfoY	T6SS1	RIMD2210633	Core activator (with Tmk). Deletion abolishes antibacterial activity. Overexpression bypasses surface sensing/salinity requirements.	(72)
RcpA	T6SS1	RIMD2210633	Positively regulates T6SS1 genes (e.g., *tssE1*).	(73)
CalR	T6SS2	RIMD2210633	Key positive regulator. Directly binds promoters of T6SS2 operons to activate transcription and antagonize H-NS.	(74)
RpoN	T6SS2	RIMD2210633	Positively regulates expression by directly binding *hcp2* and *vpa1044* promoters.	(75)
LtrA	T6SS2	RIMD2210633	Positively regulates 19 T6SS2 genes.	(76)
AcfA	T6SS2	RIMD2210633	Deletion significantly downregulates 14 T6SS2 genes.	(77)
BarA/UvrY	T6SS1/2	RIMD2210633	Deletion of *uvrY* or *barA* reduces T6SS1/2 expression 2-10 fold. UvrY directly binds *vp1407* promoter.	(48)
VPA1045/VPA1049	T6SS2	RIMD2210633	Promote Hcp2 transmembrane translocation post-translationally. Deletion weakens secretion and adhesion.	(78)
*E. coli* co-culture	T6SS2	RIMD2210633	Ecological interaction specifically upregulates 23 T6SS2 genes.	(79)
Negative regulators				
H-NS	T6SS1	RIMD2210633	Global repressor. Inhibits T6SS1 without surface sensing. Deletion increases Hcp1 expression/secretion. Inhibition is relieved by surface sensing/high salt.	(26, 71)
AcsS	T6SS2	RIMD2210633	Indirectly inhibits T6SS2 genes. Deletion enhances HeLa cell adhesion (T6SS2-dependent).	(80)
QseC	T6SS	Vp_AHPNA_123	Negatively regulates T6SS. Deletion upregulates 22 T6SS genes.	(31)

TfoY, a conserved GTPase-like regulator in vibrios, stands out as a central master activator of T6SS1 in *V. parahaemolyticus* (72). Its role mirrors its function in *V. cholerae*, where it also activates T6SS genes (81), suggesting an evolutionarily conserved regulatory module for controlling T6SS in response to overlapping environmental cues. In *V. parahaemolyticus*, TfoY overexpression can bypass the requirement for surface sensing and high salinity for T6SS1 activation, positioning it downstream of these signals (72). The regulatory hierarchy places negative regulators OpaR and H-NS upstream and downstream of TfoY, respectively, forming a coherent feed-forward loop to ensure precise activation (72). RcpA also positively regulates T6SS1-related genes such as *tssE1* (73). In the Vp_AHPND_ 123 strain, QseC negatively regulates T6SS, and deletion of *qseC* upregulates 22 T6SS genes (31).

For T6SS2, CalR is a key positive regulator that directly binds to the promoters of *vpa1027-1024*, *vpa1043-1028*, and *vpa1044-1046* to activate transcription and antagonize H-NS-mediated repression (74). RpoN positively regulates gene expression by directly binding the promoters of *hcp2* and *vpa1044* (75). LtrA positively regulates 19 T6SS2 genes (76). Deletion of *acfA* results in significant downregulation of 14 T6SS2 genes (77). In the two-component system BarA/UvrY, deletion of *uvrY* or *barA* reduces the expression of T6SS1 and T6SS2 by 2–10 times, and UvrY directly binds the promoter of *vp1407* (48). VPA1045 and VPA1049 promote the transmembrane translocation of Hcp2 at the post-translational level, and their deletion weakens secretion and bacterial adhesion (78). In terms of negative regulation, AcsS indirectly represses the transcription of T6SS2 genes (such as *hcp2*, *vpa1043*, and *vpa1044*) (80). Deletion of *acsS* enhances adhesion to HeLa cells, and this phenotype is dependent on the function of T6SS2 (80). Additionally, when co-cultured with *E. coli*, 23 T6SS2-related genes are specifically upregulated; for example, *vgrG* expression increased 7.86-fold and *hcp2* expression increased 11.28-fold, indicating that ecological interactions can activate this system (79).

In summary, the regulatory network of T6SS in *V. parahaemolyticus* is not a simple linear pathway but a highly interconnected web. Environmental cues like salinity and temperature are transduced through master regulators like H-NS and TfoY, which are in turn modulated by global systems like QS (Section 6.6) and c-di-GMP signaling. This intricate arrangement allows for the precise, condition-specific deployment of T6SS1 and T6SS2, balancing the energetic cost of these nanomachines with their benefits for competition and virulence. Future work should focus on identifying the missing links, particularly the thermosensor for T6SS1, and elucidating the molecular cross talk that allows for the seamless integration of these diverse signals.

## CONCLUSIONS

In summary, this review synthesizes the critical roles and intricate regulation of T6SSs in *V. parahaemolyticus* pathogenicity and environmental adaptation. T6SS1, enriched in clinical isolates, is activated under high-salt and warm conditions and drives antibacterial competition and adhesion to human cells. In contrast, T6SS2, nearly ubiquitous across strains, operates optimally under low-salt and low-temperature conditions and contributes to adhesion, biofilm formation, motility, macrophage autophagy, and virulence. Both systems deploy diverse effectors (e.g., DNases, nucleases, and membrane disruptors) targeting bacterial or eukaryotic cells, often paired with dedicated immunity proteins. Their expression is governed by a sophisticated multi-layered network integrating environmental cues (e.g., salinity, temperature, metal ions), chemical stresses (e.g., antibiotics, bile acids), QS, and transcription factors (e.g., H-NS, TfoY, and CalR). Significant functional heterogeneity exists across strains, reflecting variations in effector repertoires and regulatory circuitry, underscoring the complexity of T6SS biology in this pathogen.

Future research should prioritize elucidating the enigmatic T6SS3 and T6SS4 clusters, which are rare and functionally uncharacterized. Deeper mechanistic insights are needed into how T6SS effectors subvert host processes—such as VgrG2-induced autophagy—and contribute to disease *in vivo*. Such work will require advanced infection models beyond *C. elegans*, which lacks key features of the natural host environment, including physiologically relevant immune responses, host-specific metabolism, and adaptive co-evolutionary interactions. Unraveling the molecular cross talk among overlapping regulatory signals (e.g., QS, divalent cations, and bile acids) will further clarify how T6SS activity is finely tuned during infection and in environmental settings. Therapeutically, T6SS regulators and effector-immunity pairs offer promising targets for novel anti-virulence strategies. Addressing these gaps will not only advance fundamental understanding of *V. parahaemolyticus* pathogenesis but also accelerate the development of targeted intervention against this resilient seafood-borne pathogen.
